# Supplementary material for: Evaluation of Chemical Compositions and the Antioxidant and Cytotoxic Properties of the Aqueous Extract of Tri-Yannarose Recipe (Areca catechu, Azadirachta indica, and Tinospora crispa)
Source: Antioxidants (Basel). 2023 Jul 15;12(7):1428. doi: 10.3390/antiox12071428 (PMC10376335; doi:10.3390/antiox12071428)
Supplement: Supplementary file 1 [file antioxidants-12-01428-s001.zip › antioxidants-2464546-supplementary.pdf]

Supplementary material

Table S1. The mass spectra of compound identification of Tri-Yannarose recipe's aqueous extract identified via LC-QTOF-MS in the positive and negative mode based on the match scores obtained during the library search.

No.	Polarity	Name of compound identification	Mass spectra
1	Positive	13E-Docosenamide	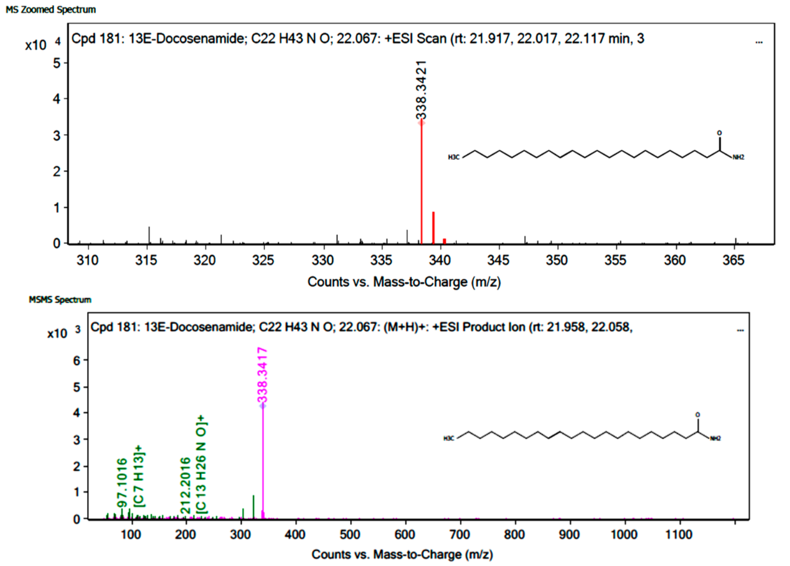

No.	Polarity	Name of compound identification	Mass spectra
			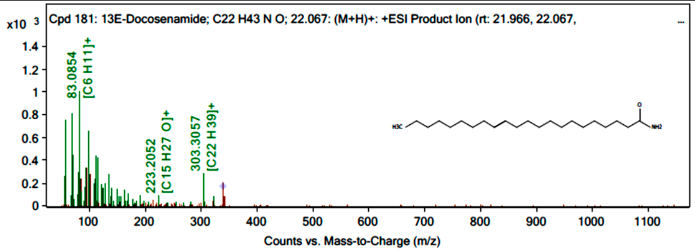<br>MSMS Spectrum<br>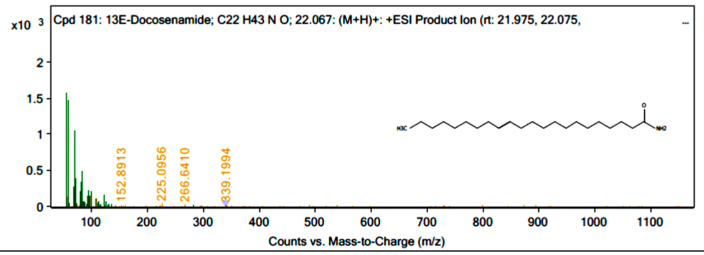
2		Choline	MS Zoomed Spectrum<br>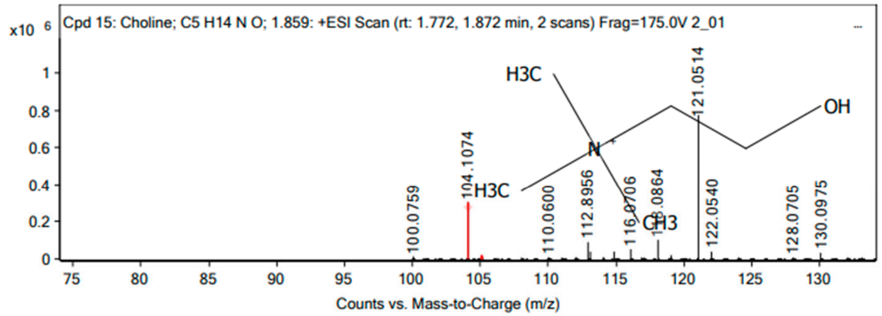<br>MSMS Spectrum<br>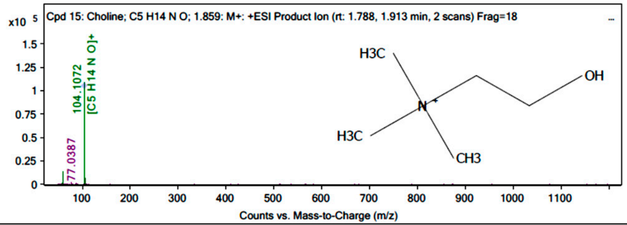

No.	Polarity	Name of compound identification	Mass spectra
			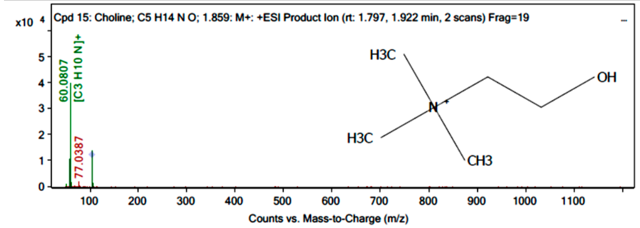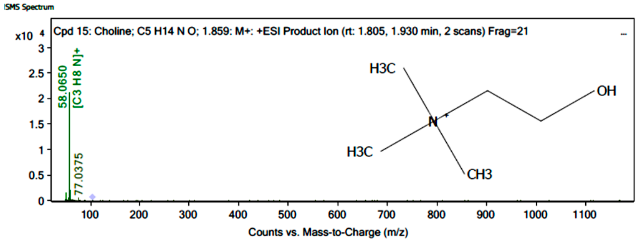
3		Guanine	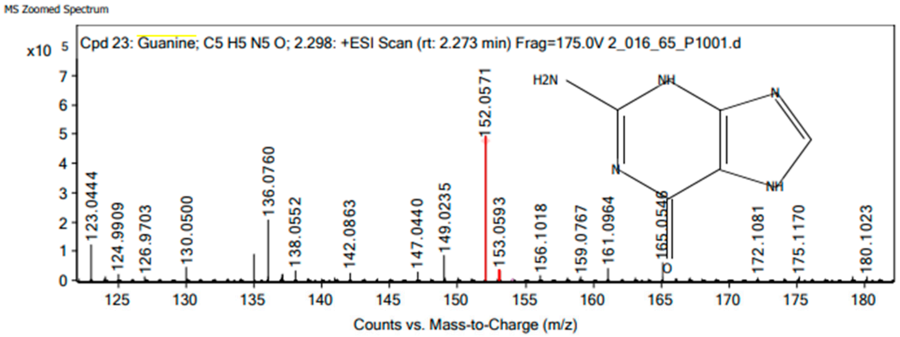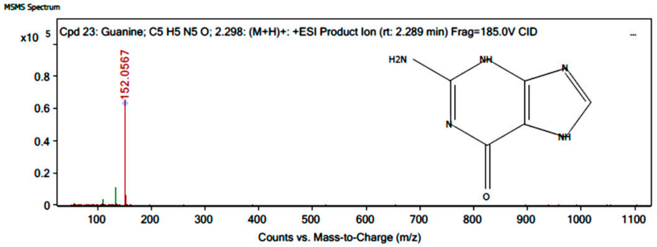

No.	Polarity	Name of compound identification	Mass spectra
			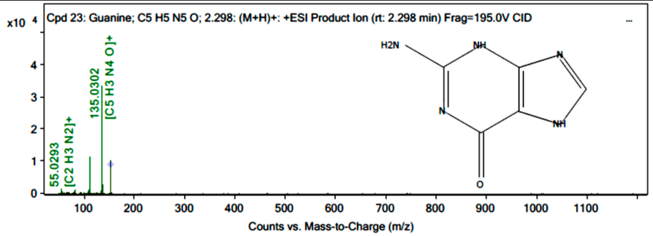 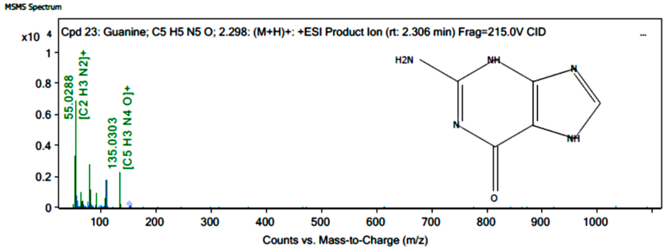
4		Trigonelline	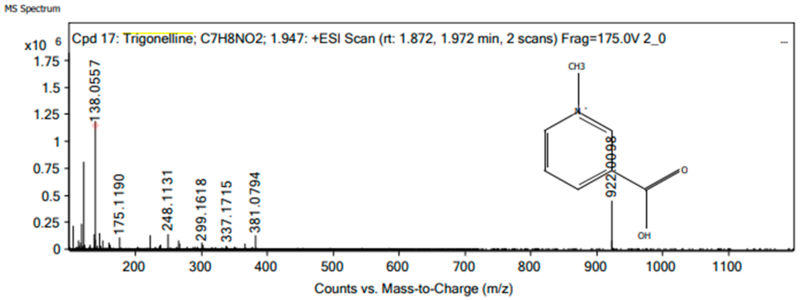

No.	Polarity	Name of compound identification	Mass spectra
			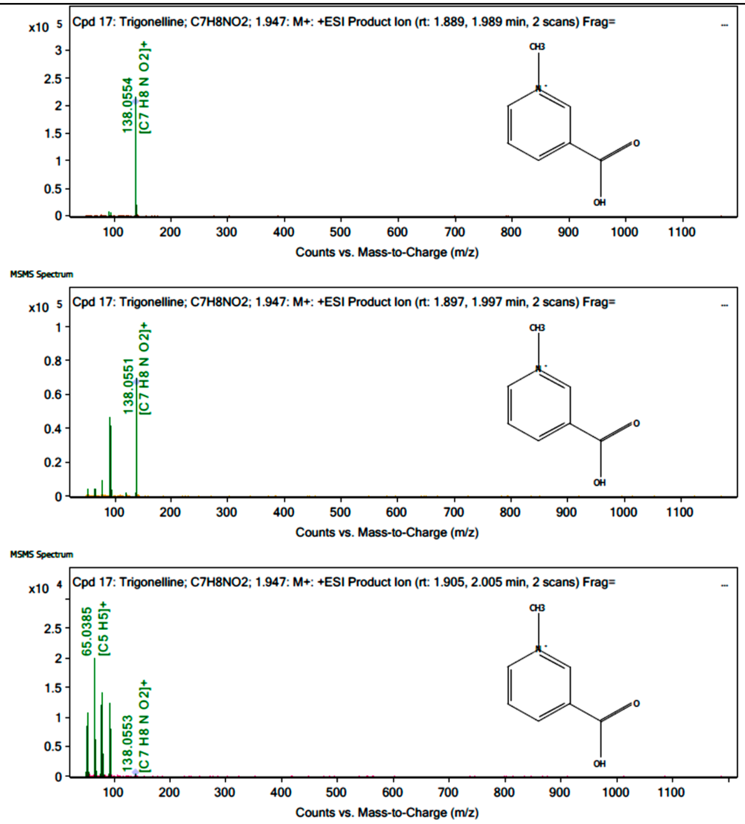

<table>
<tr><th>No.</th><th>Polarity</th><th>Name of compound identification</th><th>Mass spectra</th></tr>
<tr><td>5</td><td></td><td>Dl-tetrahydropalmatine</td><td>
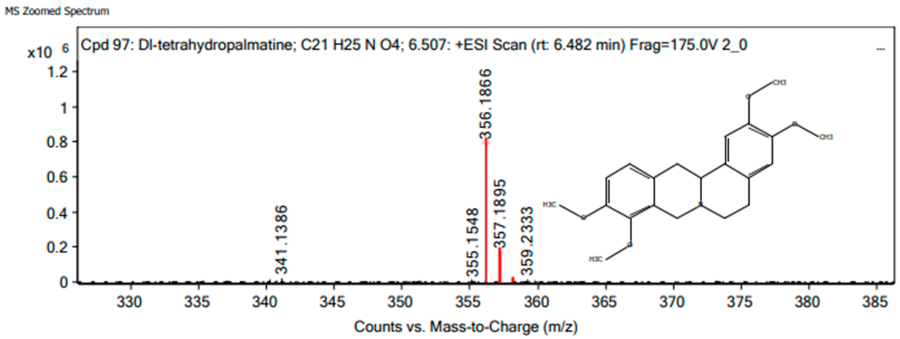

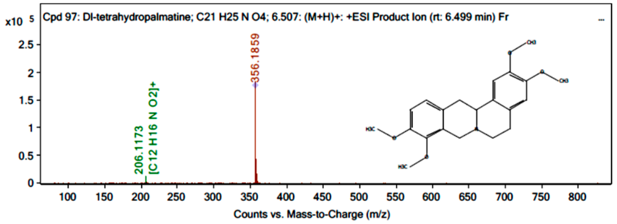

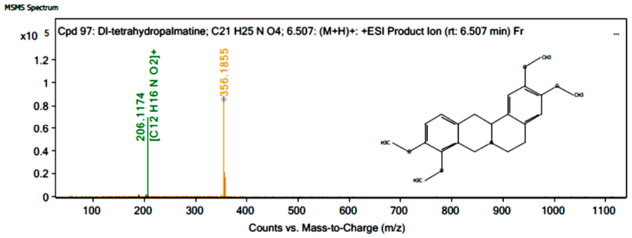

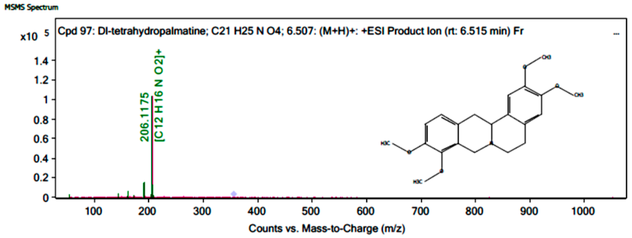

</td></tr>
</table>

<table>
<tr><th>No.</th><th>Polarity</th><th>Name of compound identification</th><th>Mass spectra</th></tr>
<tr><td>6</td><td></td><td>Phytosphingosine</td><td>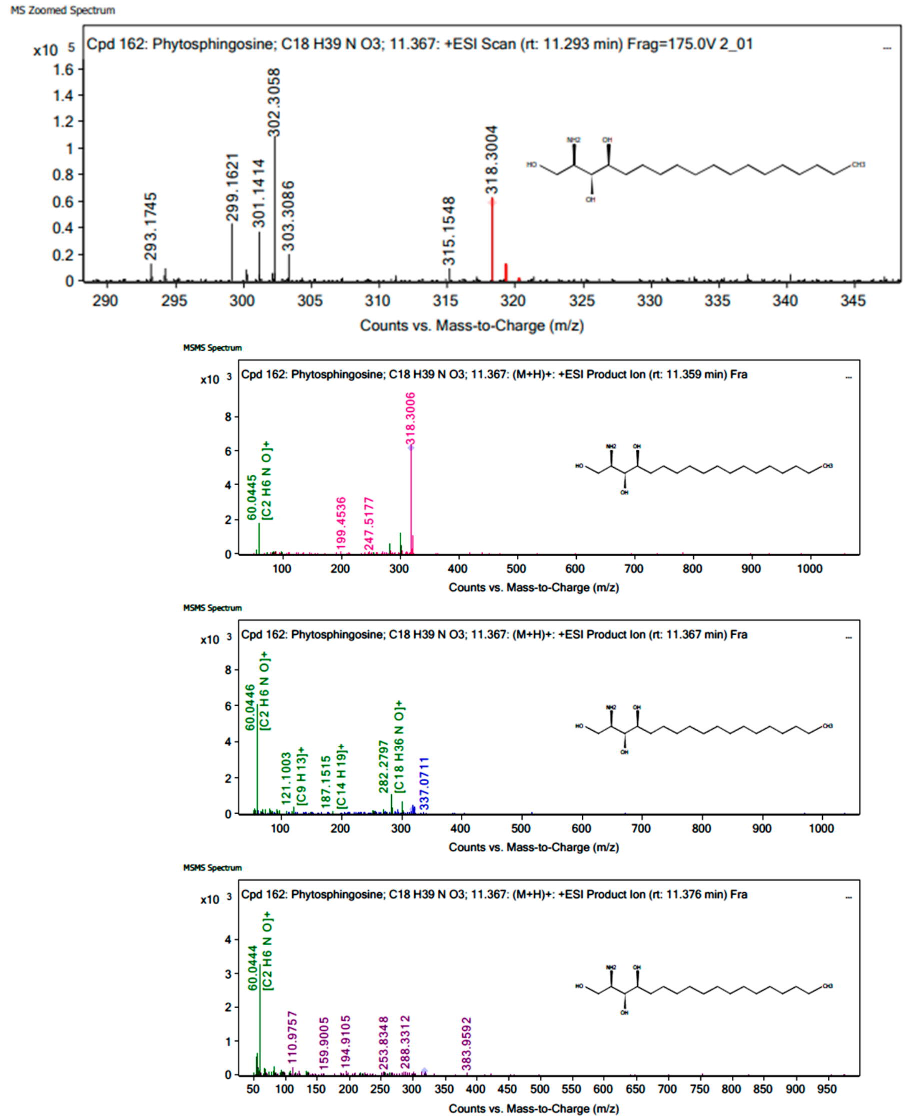
</td></tr>
</table>

No.	Polarity	Name of compound identification	Mass spectra
7		4-Hydroxycoumarin	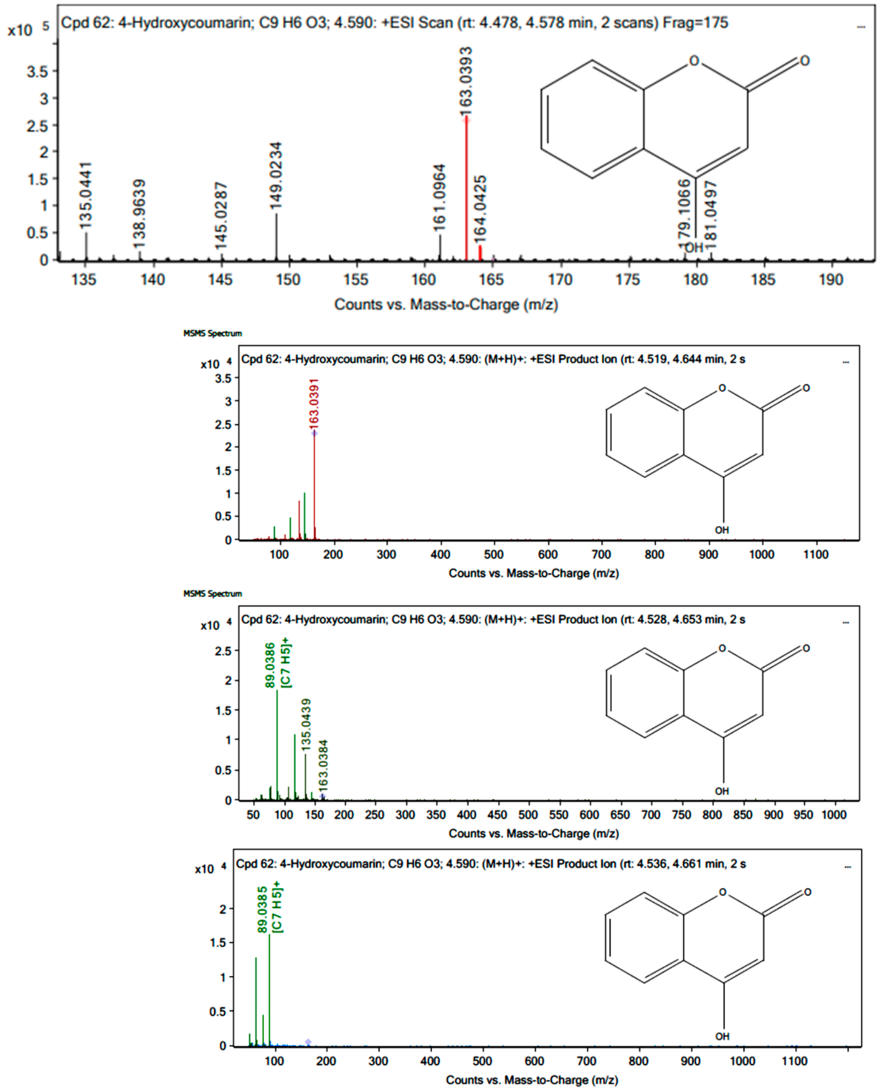

<table>
<tr><th>No.</th><th>Polarity</th><th>Name of compound identification</th><th>Mass spectra</th></tr>
<tr><td>8</td><td></td><td>Marmesin</td><td>
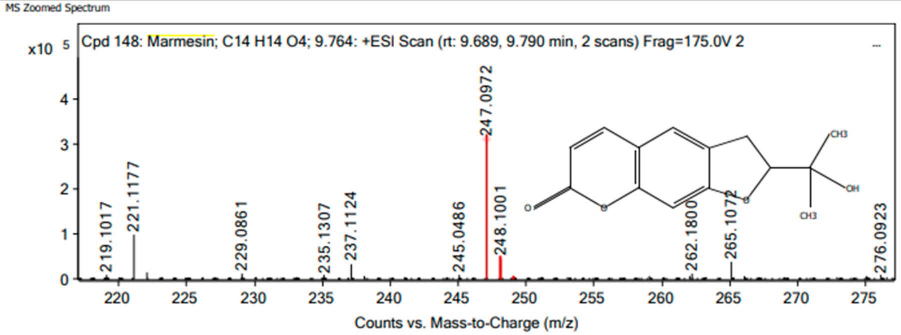

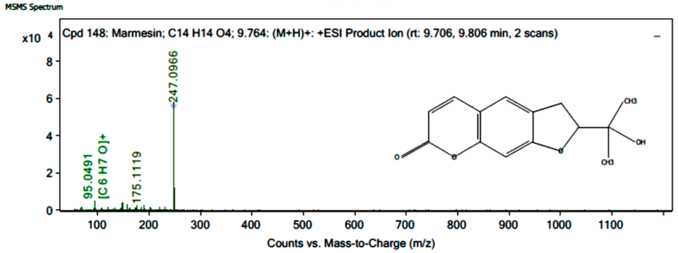

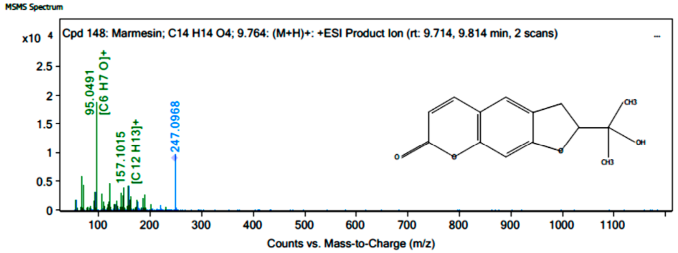

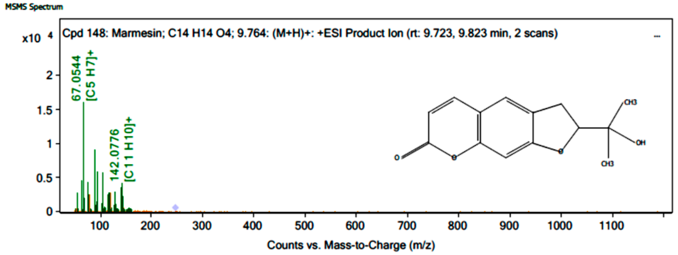

</td></tr>
</table>

No.	Polarity	Name of compound identification	Mass spectra
1	Negative	Succinic acid	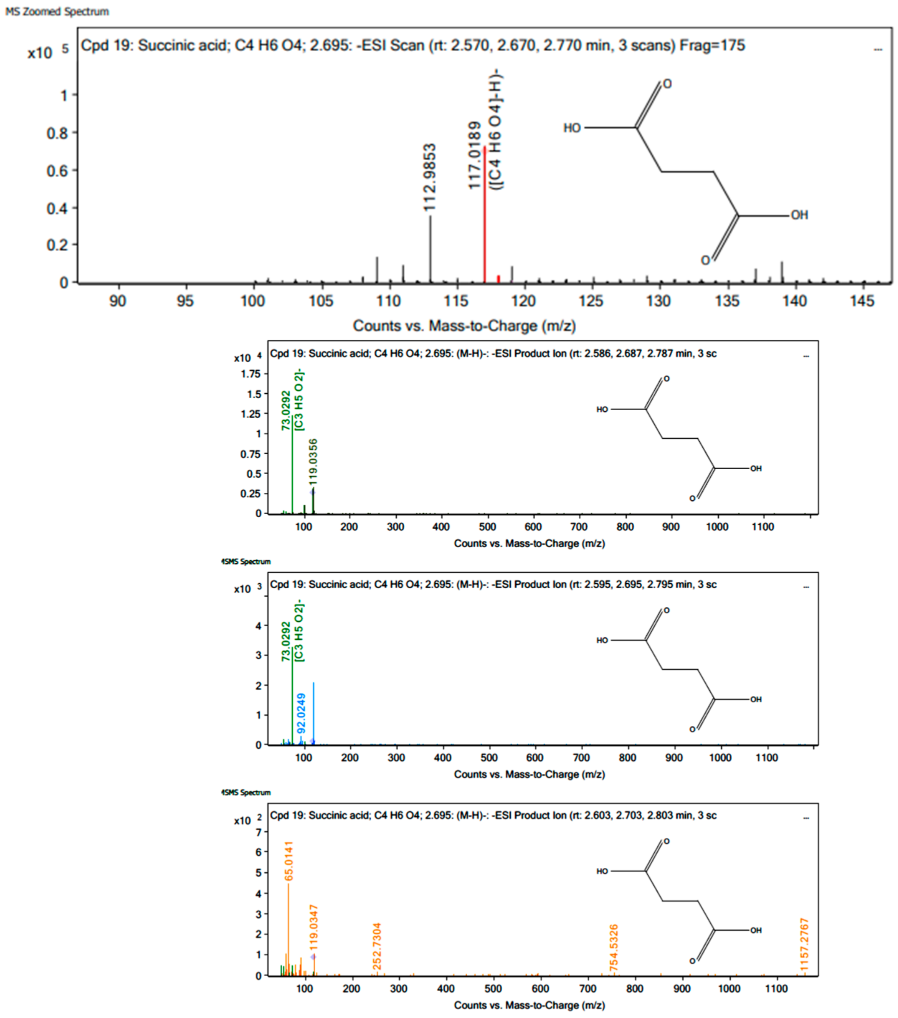

No.	Polarity	Name of compound identification	Mass spectra
2		L-α-Hydroxyisovaleric acid	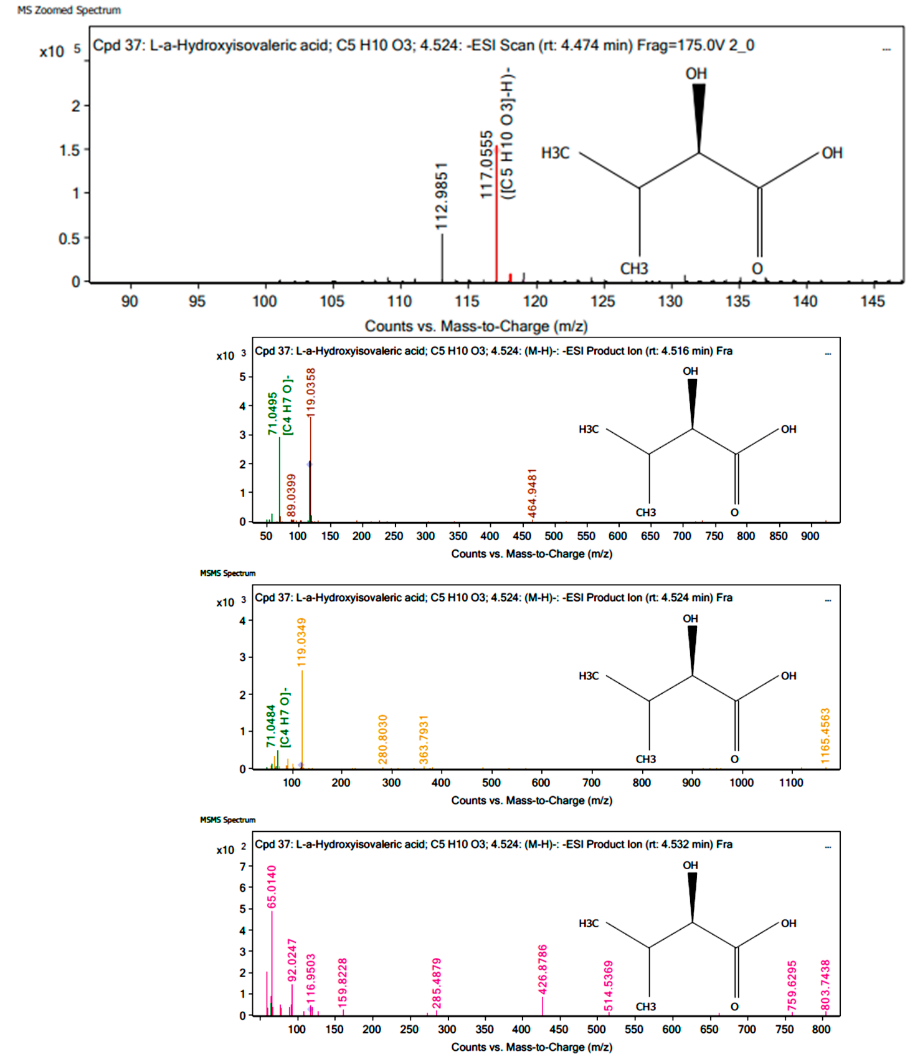

No.	Polarity	Name of compound identification	Mass spectra
3		Citric acid	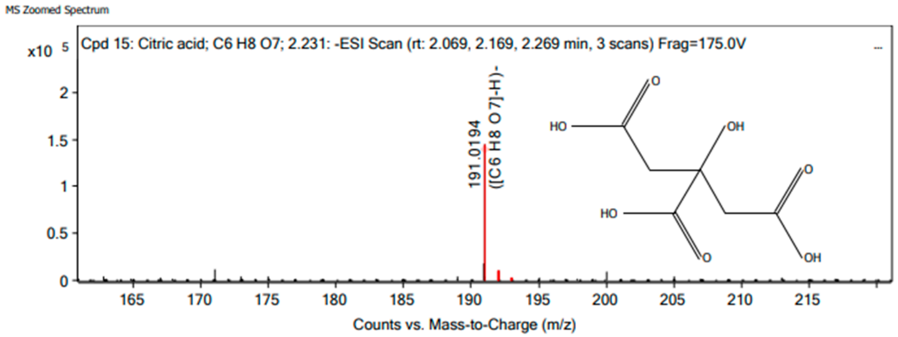 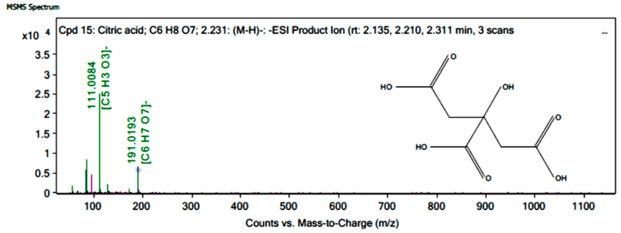 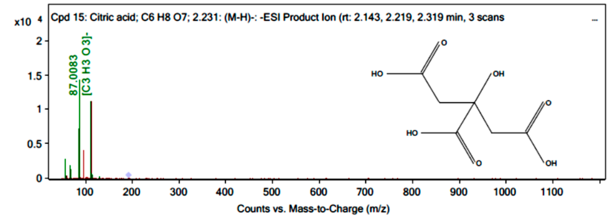 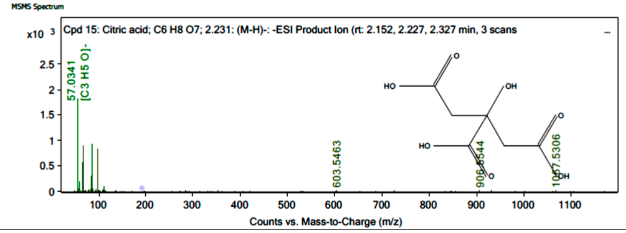

No.	Polarity	Name of compound identification	Mass spectra
4		Epicatechin	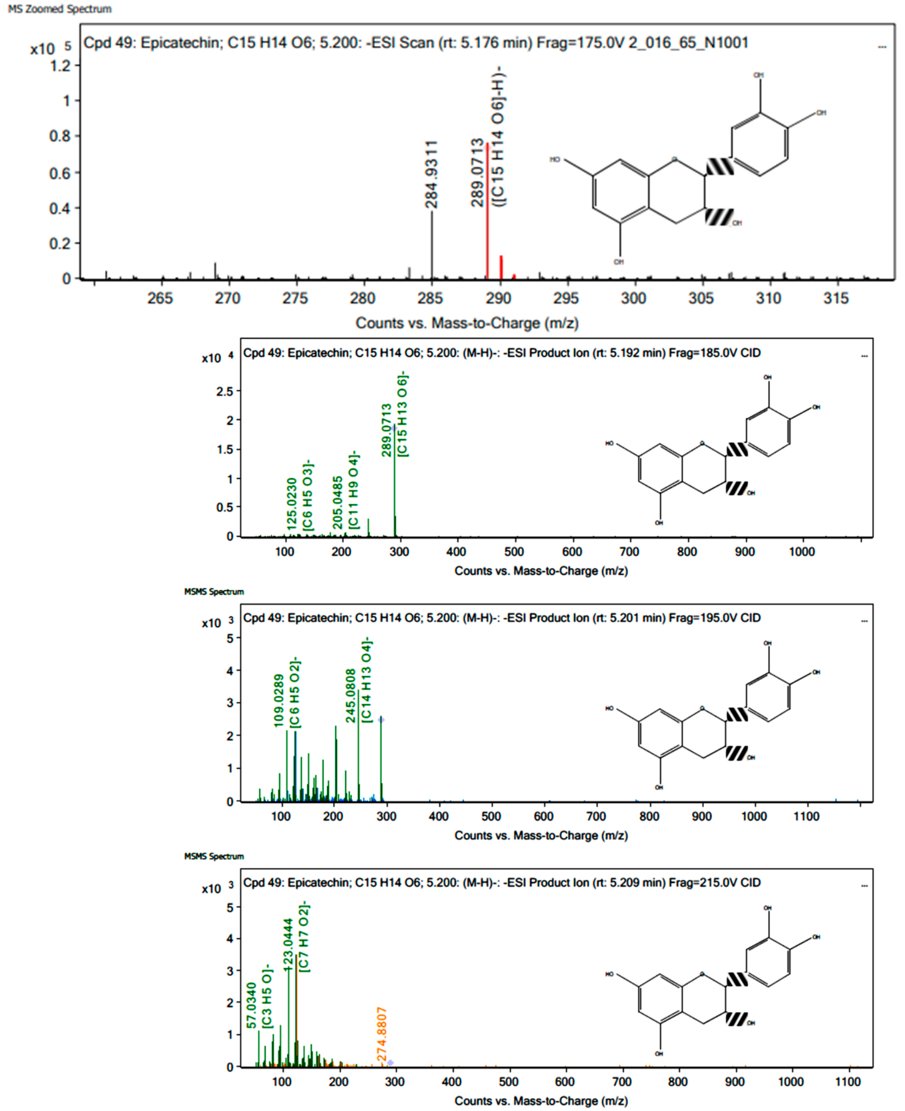

No.	Polarity	Name of compound identification	Mass spectra
5		Methyl N-(a-methylbutyryl) glycine	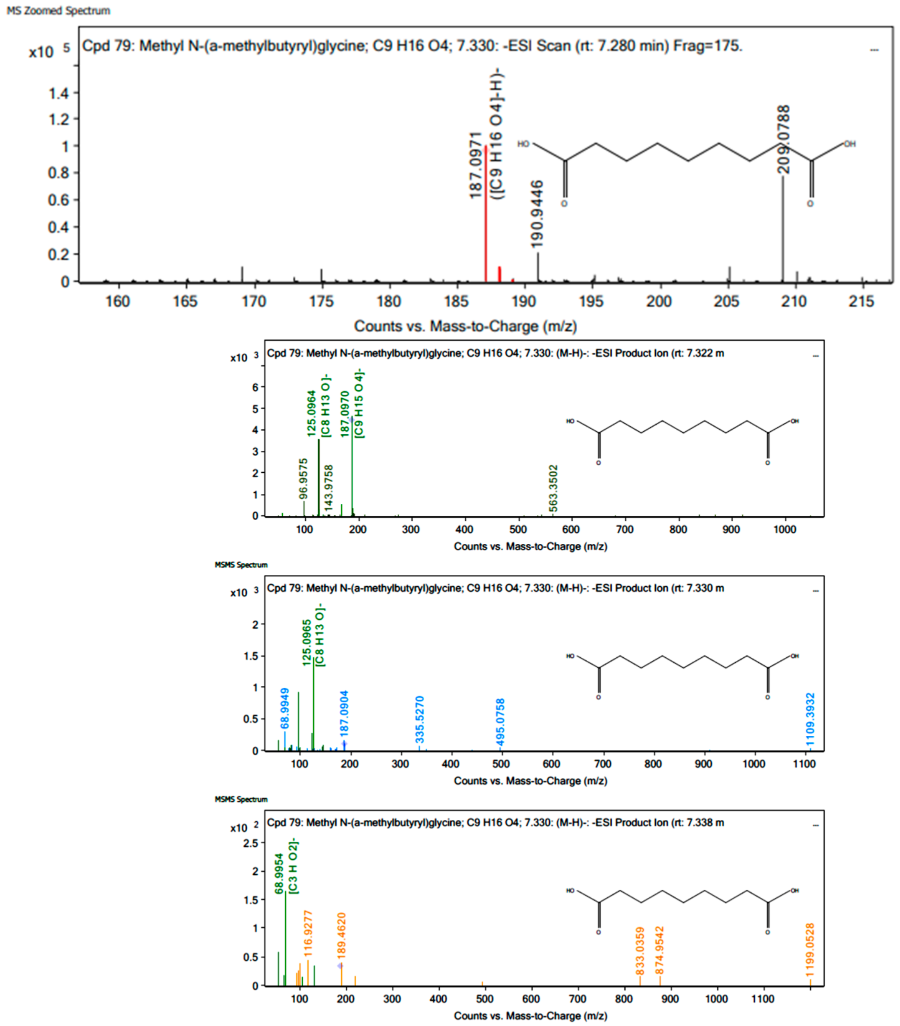

No.	Polarity	Name of compound identification	Mass spectra
6		3-Dehydroshikimic acid	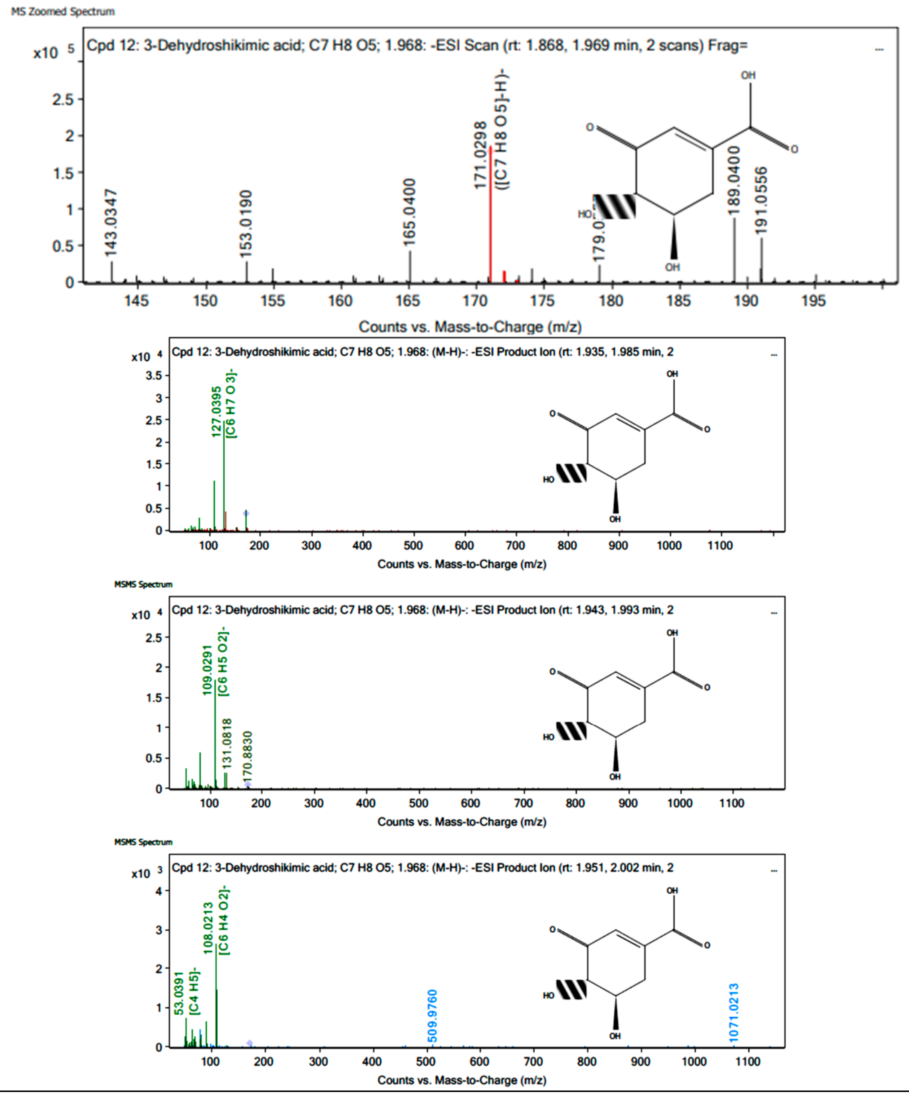

No.	Polarity	Name of compound identification	Mass spectra
7		3,4-Dihydroxybenzaldehyde	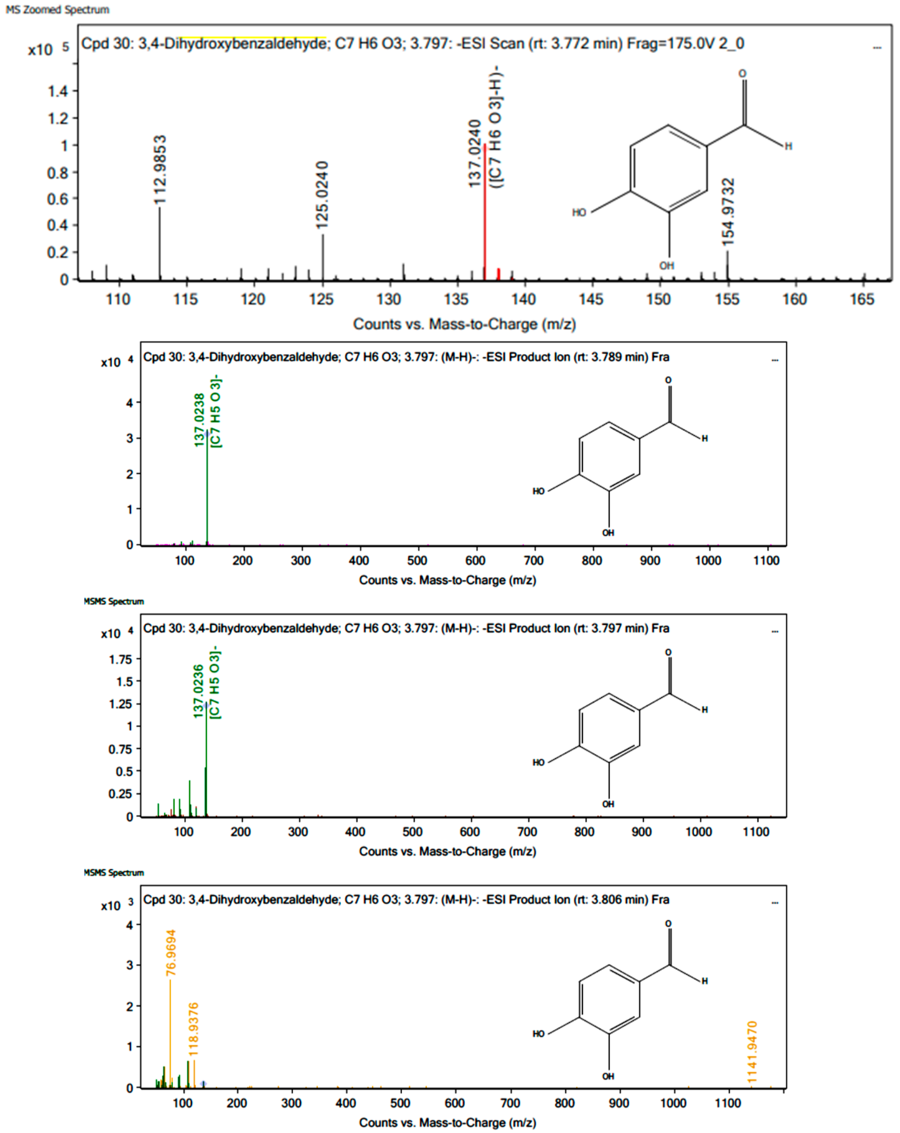

No.	Polarity	Name of compound identification	Mass spectra
8		1,2,3-Trihydroxybenzene	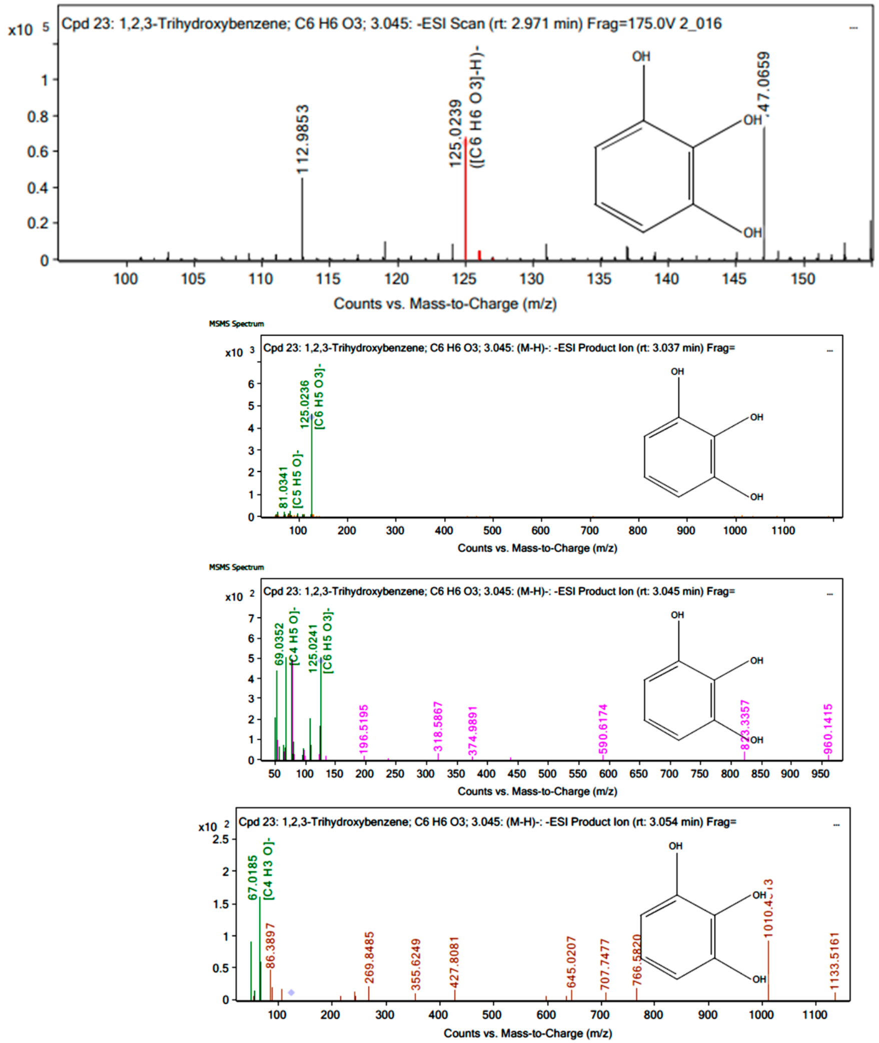

No.	Polarity	Name of compound identification	Mass spectra
9		Quinic acid	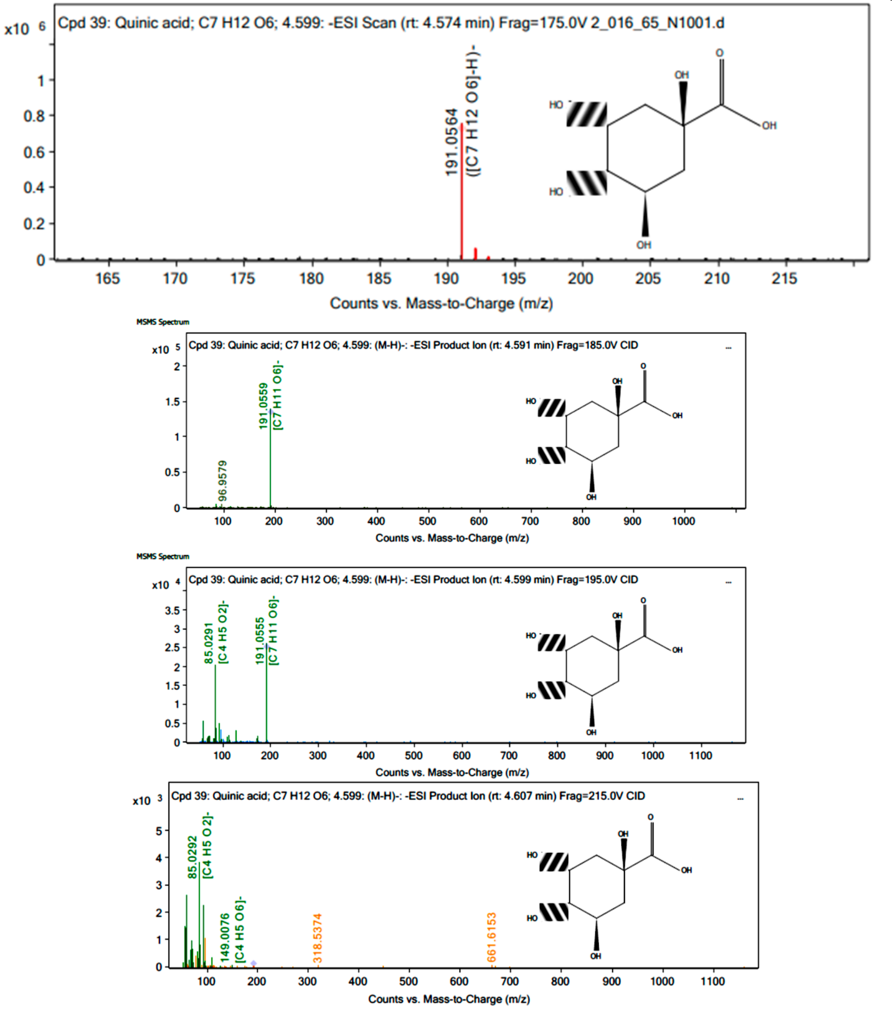

No.	Polarity	Name of compound identification	Mass spectra
10		D-(+)-3-Phenyllactic acid	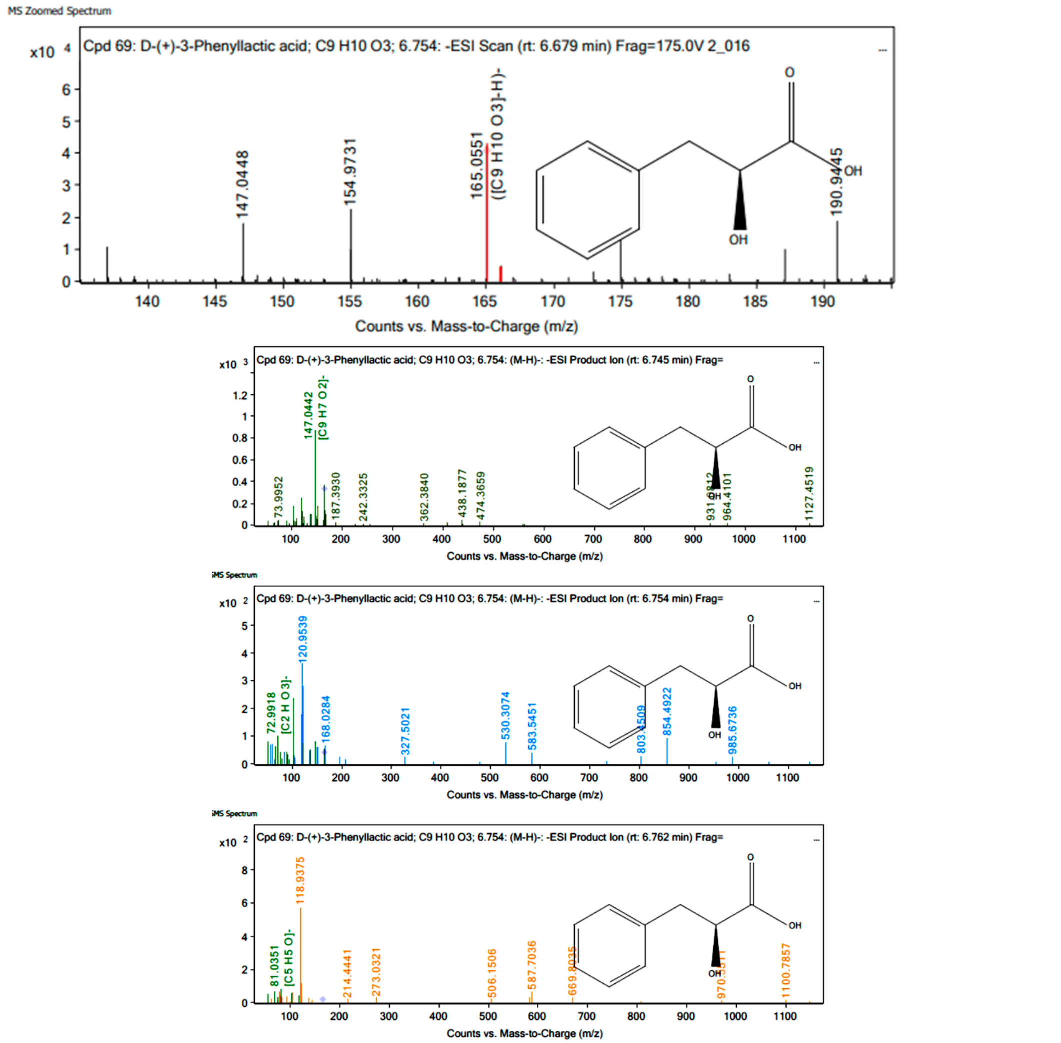

No.	Polarity	Name of compound identification	Mass spectra
11		Pyrocatechol	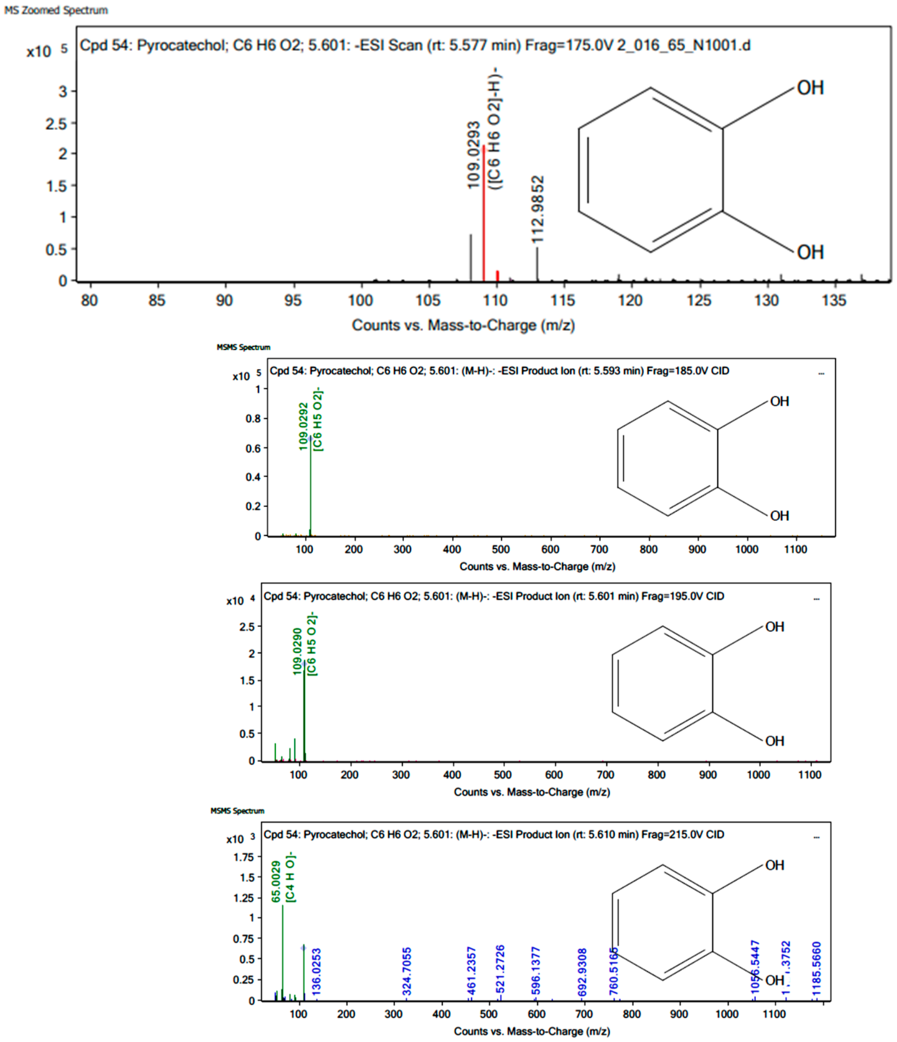

<table>
<tr><th>No.</th><th>Polarity</th><th>Name of compound identification</th><th>Mass spectra</th></tr>
<tr><td>12</td><td></td><td>Nordihydroguaiaretic Acid</td><td>
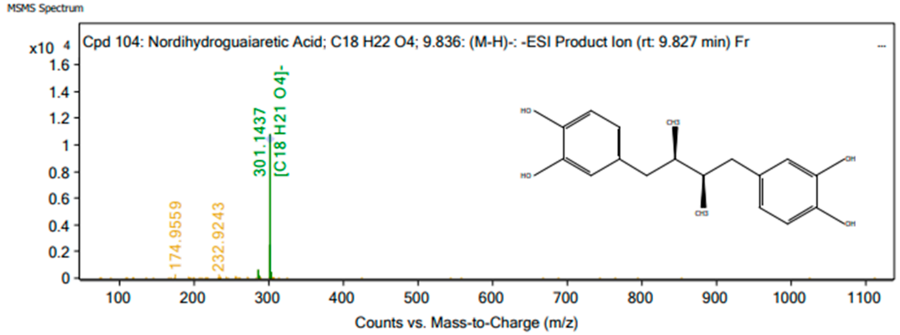

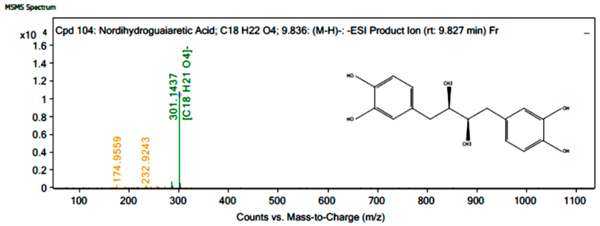

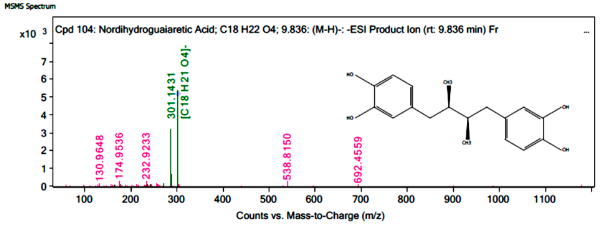

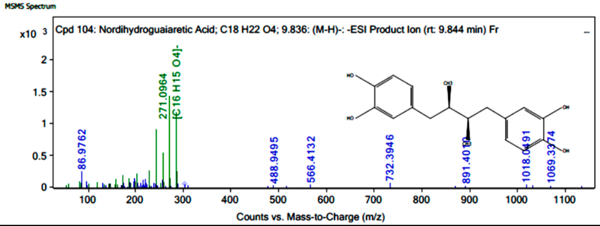

</td></tr>
</table>

No.	Polarity	Name of compound identification	Mass spectra
13		D-Ornithine	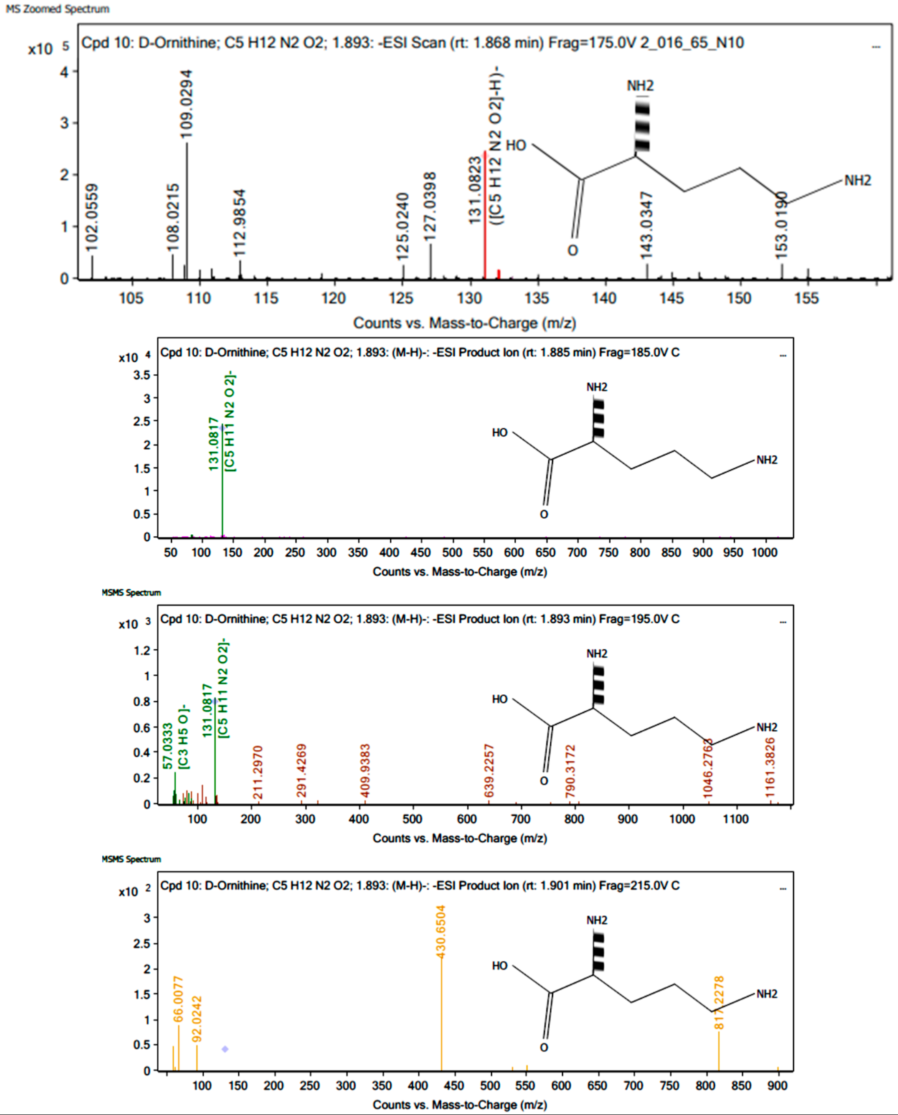

<table>
<tr><th>No.</th><th>Polarity</th><th>Name of compound identification</th><th>Mass spectra</th></tr>
<tr><td>14</td><td></td><td>Isopalmitic acid</td><td>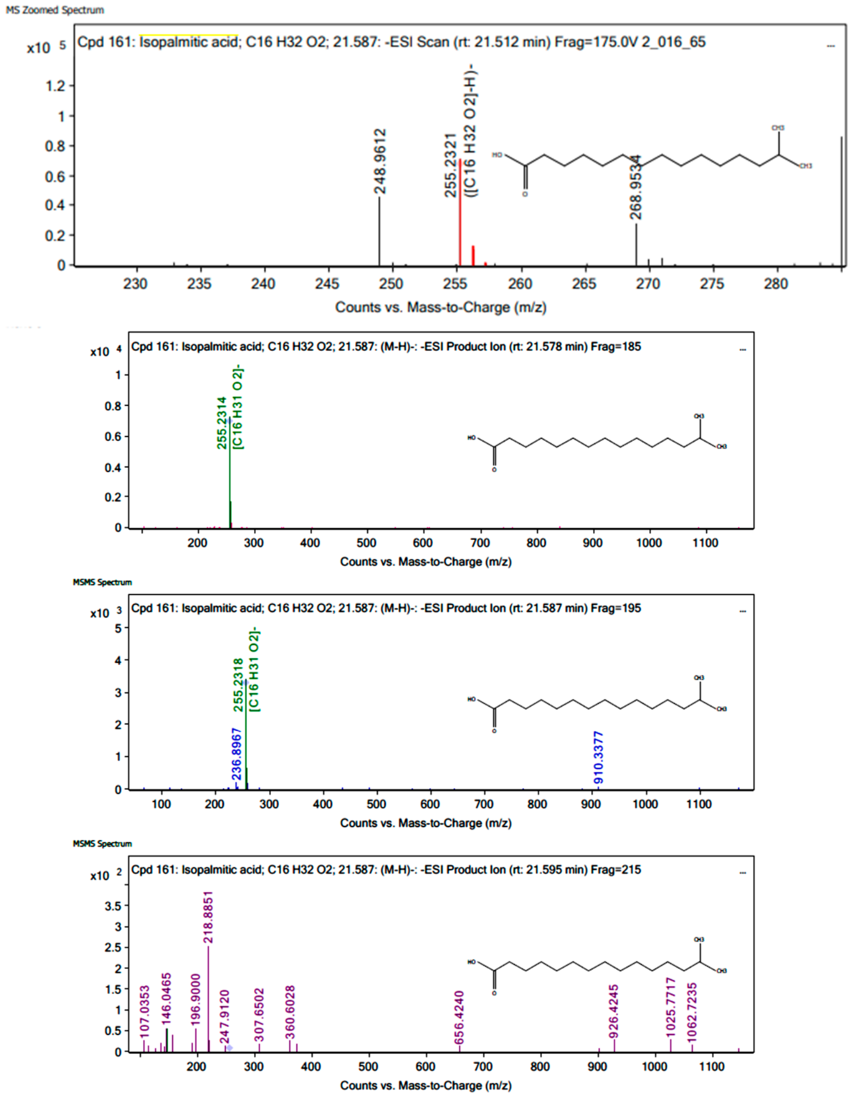
</td></tr>
</table>
